# Gender-Based Violence Against Trans* Individuals: A Netnography of Mary Gregory’s Experience in Powerlifting

**DOI:** 10.3389/fpsyg.2022.854452

**Published:** 2022-05-06

**Authors:** Raiya Taha-Thomure, Aalaya S. Milne, Emma J. Kavanagh, Ashley E. Stirling

**Affiliations:** ^1^Faculty of Kinesiology and Physical Education, University of Toronto, Toronto, ON, Canada; ^2^Faculty of Health and Social Sciences, Bournemouth University, Poole, United Kingdom

**Keywords:** gender, microaggressions, maltreatment, interpersonal violence, cyberculture

## Abstract

In the context of sport, a growing body of research has reported the prevalence of violence against athletes, including sexual, physical, and psychological violence and neglect, experienced by both women and men in sport. Preliminary research has reported that gender-diverse individuals, specifically transgender athletes, may have a greater vulnerability to experiences of violence in sport, but this remains an under-researched population. In addition to limited research specifically on violence experienced by transgender athletes in sport, there is also only emerging research on virtual violence against athletes, with previous research on virtual violence in sporting spaces highlighting how online spaces are sites that can foster widespread hostility and violence. This study builds on previous research by examining discourses of virtual violence faced by transgender powerlifter, Mary Gregory, following her expulsion from the 100% Raw Powerlifting Federation. This research used a netnographic approach—an online ethnographic case study design. Data were collected from online news sources, as well as social media platforms, including Instagram, Twitter, and YouTube and were analyzed using reflexive thematic analysis. The data provided an insight into the cyberculture of powerlifting, and the negotiation of space, or lack thereof, for Mary Gregory within this physical culture. Five themes of were generated, including invalidation of gender identity, dehumanization, infliction of derogatory and crude language, accusations of cheating, and being compared to cisgender athletes without nuance. The study highlights the presence of significant vitriol across virtual platforms directed at Mary Gregory and the underlying presence of negative gender-based violence again trans* (GBV-T*) discourse. This case provides examples of virtual gender-based violence and transphobia in sport, a lack of readiness to accept trans* athletes, and concerns for the safety of trans* athletes in sporting spaces.

## Introduction

### Background Literature

Violence, defined by the World Health Organization (WHO) as, “the intentional use of physical force or power, threatened or actual, against ones-self, another person, or against a group or community, that either results in or has a high likelihood of resulting in injury, death, psychological harm, maldevelopment or deprivation” (Kilpatrick, 2004, p. 1213), has been a topic of growing research attention in sport.

In the context of sport, a growing body of research has reported the prevalence of violence against athletes, including sexual, physical and psychological violence and neglect, experienced by both women and men in sport (Parent and Vaillancourt-Morel, 2020; Ohlert et al., 2021; Willson et al., 2021). Preliminary research by Vertommen et al. (2016) aimed to capture the experiences of equity deserving populations, and reported that gender-diverse individuals, specifically transgender athletes, may have a greater vulnerability to experiences of violence in sport, but this remains an under-researched population.

Complementary research on the inclusion of transgender athletes as reported that the presence of transgender athletes in sport challenges hegemonic beliefs grounded in a binary categorization of sex and gender, which recognizes women and men as categorically different. Individuals falling somewhere between these binary categories often face social repercussions, discrimination and violence in sport (Krane, 2014). Lucas-Carr and Krane (2011) highlighted how strict adherence to this dichotomy has resulted in the exclusion and stigmatization of transgender athletes, including the perpetuation of anti-trans rhetoric. Additional barriers to participation of transgender athletes in sport include changing and showering facilities, as well as standard sport clothing (Jones et al., 2016). The constant fear surrounding the safety for transgender people, as well as the exclusive cultures of sport, is a recipe for violence, discomfort, and low sport participation (Jones et al., 2017).

Recent attention in academic literature has also described the social and legal difficulties transgender athletes can encounter in pursuit of equity and equality in sport. Inequities and policy-based discrimination against transgender people in sport exist on the commonly held belief that there is an inherent unfair advantage held by transgender athletes, especially trans-women, above cisgender athletes (athletes whose gender corresponds with their birth sex), on the basis of hormone discrepancies (Jones et al., 2017; Colliver, 2021). Such a belief is fed by cisnormative and binary understandings of human physiology, doping, and hormone supplementation, tied to discourses of cheating and “unfairness” likened to the mere existence of transgender athletes (Tominey, 2019; Sailors, 2020). Natural genetic differences in hormone levels, mainly focused on androgen levels, has become the qualifier or disqualifier for competitive athletes, becoming a key point in the debate against transgender athletes, and trans-women specifically (Jones et al., 2016).

In addition to limited research specifically on violence experienced by transgender athletes in sport, there is also only emerging research on virtual violence against athletes (Kavanagh et al., 2016; MacPherson and Kerr, 2021). Previous research on virtual violence in sporting spaces highlights how online spaces are sites that foster widespread hostility and violence (Kavanagh et al., 2016). In many ways virtual worlds mirror face-to-face environments, therefore violence which is present in physical spaces can be replicated in and/or augmented by online environments (Litchfield et al., 2018; Kavanagh et al., 2019). Athletes can become a target of virtual violence for a multitude of reasons. For example, in order to publicly shame an athlete due to a norm violation (MacPherson and Kerr, 2021), as a result of speaking out concerning a social or political issue that diverges from how a fan feels about that topic (Sanderson et al., 2016; Frederick et al., 2017), or when an athlete outperforms others and is viewed as an outlier (Litchfield et al., 2018). Furthermore, Litchfield et al. (2018) highlighted the presence of sexist, racist and violent interactions toward women athletes in virtual spaces enacted by sports fans or followers of sports on the basis of the athlete’s gender (Kavanagh et al., 2021).

No research exists specifically on experiences of virtual violence against transgender athletes in sport. This study builds on previous research by focusing specifically on virtual violence experienced by transgender athlete Mary Gregory, a previously unexplored dimension of online social discourse.

### The Case of Mary Gregory

The case of Mary Gregory, male-to-female (MTF) transgender powerlifter is the focus of this research. This is a noteworthy case of investigation as the removal of Mary Gregory’s titles following the 100% RAW Powerlifting Federation competition carries great importance as one of the most publicized instance of transgender discrimination in the sport of powerlifting.

USA Powerlifting (USAPL) is the largest and most popular powerlifting federation in the United States. In 2019, the USAPL made clear statements about their zero-tolerance policy toward transgender athletes, resulting in outrage, and upset for transgender powerlifters and allies (Villareal, 2019). Other federations retrospectively stripped transgender powerlifters of their titles and memberships, including trans-woman Mary Gregory. Mary Gregory competed with the federation in April of 2019 for the first time in the 100% RAW Powerlifting Federation competition, 1 year after beginning hormone replacement therapy as part of her gender affirmation treatment. On the platform, Mary Gregory’s performance was unmatched, but during the drug test, questions arose about her assigned sex at birth. This was a case where the 100% RAW Powerlifting Federation had no policies in place regarding the participation of transgender athletes. Consequently, Mary Gregory’s results were nullified and her case was made public by the federation, fueling online scrutiny and comment.

### Research Purpose

The purpose of this research was to examine the virtual violence incurred by transgender athlete Mary Gregory through online discourses following the nullification of her results by the Powerlifting Federation.

## Methodology and Methods

### Netnography

Assuming a critical relativist ontology and a critical subjectivist epistemology, the study adopted a post-structuralist paradigmatic position (Tamminen and Poucher, 2020). With the understanding that online spaces present a growing concern of violence toward athletes (Kavanagh et al., 2019), the study was conducted using a netnographic approach in which the researcher applies ethnographic procedures in online spaces (Kozinets, 2010). Netnography exists as a specialized form of ethnography, which has been adapted to capture interaction in computer-mediated environments (Kozinets, 2010). As an overarching approach, netnography enables researchers to observe and gain an understanding of the voices of individuals and collective interactions online. This form of research is an iterative process adopting naturalistic analysis techniques that are immersive in nature (Reid and Duffy, 2018). The online format of this netnography provided the researchers with a substantial information pool on the emergent online subcultures, or “cybercultures” of powerlifting, and the virtual violence directed at transgender athlete, Mary Gregory.

### Data Collection

Data collection followed Kozinets (2010) guidance on netnography, including: research planning, entrée, data collection, interpretation, ensuring ethical standards, and research representation. Archival data were collected from three online news sources (Washington Post, Pink News, RedState), as well as social media platforms (Twitter, Instagram, YouTube). Archival data allows the researcher to explore what is publicly available on the internet rather than having any researcher interaction with these online communities or spaces (Cleland et al., 2020). Firstly Google searches were conducted to identify popular media and discussion posts from various online sources around the event. Examples of search terms include “Mary Gregory,” “transgender powerlifter,” and “transgender weightlifter.” From the Google search, data were found from the Washington Post, Red State News, Pink News, and Twitter. In addition to data from the Washington Post article itself, data from the comments related to the article were collected. Similarly on social media platforms such as Twitter, subtweets or comments were used to capture running commentary. The second strategy of data collection was specific to social media applications using Instagram and YouTube to find user comments under posts. On Instagram, comments were taken directly from Mary Gregory’s personal page on posts dated within 2 weeks of the event. This timeframe was selected in order to specifically focus on online discourse directly following the nullification of Mary Gregory’s results by the Powerlifting Federation to explore immediate reaction surrounding this critical event. Commentary on YouTube videos of the event were also reviewed for collection of comments. Thousands of comments were read and sifted through. Of these comments, 109 were deemed as relevant to the purpose of this study and were reviewed in the data analysis. All comments used in the data analysis reflected a component of virtual violence toward Mary Gregory, or transgender women in general (see Table 1).

**TABLE 1:** Media source and number of comments.

Type of media source	Number of comments
YouTube	60
Twitter	16
Instagram	12
Washington post (News source)	15
RedState (News source)	6

### Data Analysis

Data were analyzed using an inductive approach to reflexive thematic analysis, outlined by Braun and Clarke (2019). The primary researcher first familiarized themselves with the data by re-reading all of the information collected. Second, codes were created by highlighting the most important findings from the data, for all of the data. Third, the researcher generated initial themes by grouping codes together based on broader similarities. Fourth, themes were reviewed to ensure they aligned with the initial research question. Fifth, the researcher named and defined the themes, in collaboration with the larger research team, including flushing out further details beneath each. Finally, the themes were analyzed and contextualized according to the present case study.

### Ethical Considerations

Although the information collected and analyzed in this research was publicly sourced and widely available through the chosen social media platforms, several steps were taken to ensure the ethical considerations of a netnography research design. Understanding that the data collected was not initially generated for research purposes, all user/author details were anonymized for the sake of safety and privacy. Usernames and identifying information have intentionally been omitted from both the research notes, as well as the manuscript itself. The only identities kept in the research are of Mary Gregory. Furthermore, there were no interactions or communication between the researcher and members of the studied cyberculture.

## Results

The publicity of Mary Gregory’s banishment from the 100% Raw Powerlifting Federation and the removal of her winning title resulted in a cascade of online discourses and commentaries. In total, 109 comments were recorded as having themes of virtual violence against Mary Gregory on the basis of her transgender identity. These comments were collected across various social media sources. While Mary Gregory was the impetus and target for the majority of comments, much of the rhetoric was aimed at transgender women and the LGBTQ + community in general. There was little difference in rhetoric between the social media platforms themselves. From comments on Mary Gregory’s personal Instagram page, to comments under news articles, to Twitter threads, the same themes of transphobia were recurrent.

The following five themes were generated regarding the virtual violence experienced by Mary Gregory within powerlifting cyberculture. These themes include, invalidation of gender identity, dehumanization, infliction of derogatory and crude language, accusations of cheating, and being compared to cisgender athletes without nuance.

### Invalidation of Gender Identity

The deliberate use of gender non-affirming language against Mary Gregory comprised the majority of virtual violence within the data. Invalidation of Mary Gregory’s identity was classified as misgendering, attempts at dead-naming, as well as the suggestion that Mary Gregory is not a “real woman” (YouTube). Misgendering was mainly categorized as using he/him/his pronouns for Mary Gregory, whose pronouns are she/her/hers.

Deadnaming is the use of a transgender person’s name assigned at birth in lieu of their chosen name. Mary Gregory’s name at birth was not available as public information, so many have chosen to call her “Gregory” as a first name instead of “Mary.” An example included a comment stating, “How does his face not fall off with shame? […] What kind of person does this? And those insane enablers calling him “Mary” (YouTube). This commenter, and many others, used a combination of pronoun misgendering, gaslighting, and attempts at deadnaming. Placing the responsibility on others for “enabling” Mary Gregory in her transgender identity suggests that her identity is an affliction that must be dealt with without support or respect (YouTube). By labeling those who merely respect transgender people as “insane,” the intended harm falls on Mary whose identity was seen as undeserving of support (YouTube). As such, this comment invalidated all transgender people by othering and painting their identities as unnatural.

Many of the comments invalidating Mary Gregory’s identity as a woman expressed a lack of empathy and understanding, citing genitalia and hormones as a determinant of gender identity. While sex and gender may be linked for some individuals, they are not necessarily related for all people. This discrepancy, and the lack of knowledge about the differences between sex and gender, produced the apparent fear and frustration toward Mary and her grasp of social identities. Additionally, it bred opportunity for deliberate attempts at misgendering on the basis of sex characteristics. One commenter, “[believes] Mary has a dick and balls,” using male anatomy as a disqualifier of her identity as a woman (YouTube).

While some individuals recognized Mary Gregory as a woman, they discredited her womanhood and refuted her identity by titling cisgender women as “real women” and “biological women” (YouTube; RedState; Washington Post; Twitter). The implication of these comments in binary opposition was that transgender women are fake women and are biological men. Such comments disguised themselves as being progressive with the use of she/her/hers pronouns and the recognition that Mary Gregory is a woman, but the backhanded under-layers of violence semantically implied were as invalidating of transgender identities as deliberately using the wrong pronouns.

### Dehumanization

While many commenters exercised their prejudice against Mary through insisting that she is a man or a fake woman, others used her transgender identity as an opportunity to dehumanize her altogether. Dehumanization can be seen in the following quote “IT didn’t win anything but praise in ITs own head!!” (Twitter). Examples of dehumanization also included the use of the inanimate it/its pronouns in lieu of the animate she/her/hers, they/them/theirs, *or* he/him/his pronouns.

The use of it/its pronouns was a deliberate attempt at politicizing Mary Gregory’s existence and dramatizing the events of this case study. Mary Gregory has stated on her Instagram page that she uses she/her/hers pronouns. By refusing to use these pronouns, or even other pronouns that people of other gender identities use, such as he/him/his or they/them/theirs, the commenter positioned Mary as sub-human or not human at all. The commenter refused the social identity of gender and resorted to externalizing violence against Mary Gregory instead of understanding her identity and experience.

Other comments such as “Wow look at that freak,” “I thought the thumbnail was for a carnival freak show,” and “Anyone else waiting for this circus to end?” indicated the dehumanization of Mary Gregory (YouTube). Such language was used to other and paint her identity as straying from the human condition. The allusion to “freak [shows]” suggests that Mary Gregory’s identity as a transgender person is comparable to and on par with health anomalies put on display by these carnivals. The perpetuation of the idea that transgender people are “freaks” and are straying from human nature is deeply harmful to both the recipient of the message and the community as a whole. This language attempted to silence and suppress transgender people by making them believe that they are not worthy of the same basic privileges held by cisgender people.

### Crude and Derogatory Language

Mary Gregory not only received malicious language through the form of pronoun/name misgendering and dehumanization, but she also endured crude and derogatory language centering her transgender identity. This included jokes about any aspect of her identity as well as slurs.

Sex-based crude directed at Mary Gregory were a common theme. Many jokes attempted to make fun of Mary Gregory by placing male/female and man/woman characteristics in binary opposition. Some examples included: “Her hairline is higher than her T levels!” “It definitely took balls to set those new records,” as well as dialogue jokes (YouTube). An example of dialogue jokes seen in comments is as follows: “Doctor: sir, Powerlifter: it’s ma’am, Doctor: ma’am u have testicular cancer” (YouTube). The goal of these jokes were to create a sense of outrage and absurdity by “mismatching” gender and sex characteristics. Within these jokes, Mary Gregory’s assumed biology was used as a punchline against her pronouns and identifiers. In the first joke, she/her/hers pronouns were used in opposition to Mary Gregory’s testosterone levels. The perpetuation of misinformation surrounding the medical transitions of transgender people fed into the echo chamber of transphobic and anti-trans sentiment in sport physical cultures.

In the third joke listed above, the epithet “ma’am” was used in opposition to testicular cancer. Common transmisogynistic rhetoric include jokes about the “woman with a penis.” Here, the intersectional role of the transgender identity and the woman identity created a joke on the basis of what constitutes womanhood. Women are often understood through a patriarchal system and their relation to men. In a patriarchal system, men are seen as penis-having, strong, and dominant. Consequently, women are penis-lacking, weak, and subservient in their positionality to men. Mary Gregory’s identity and assumed anatomy rejected the patriarchy’s hegemonic prescriptions of gender roles. It is possible that cisgender heterosexual (cishet) society uses crude humor as an attempt at understanding the trans spectrum and its differences from cishet roles. Even so, the impact of these jokes are greater than their intention. It should be noted that some of the language was filtered using online algorithms, which barred comments due to their content, therefore the full extent of the language adopted cannot be captured.

### Accusations of Cheating

A set of discourses employed by commenters to diminish the accomplishment of trans-women athletes comes through accusations of cheating. Across every single social media platform reviewed, there were comments such as “cheater” and the suggestion that Mary Gregory was stealing awards from “biological women” (YouTube; Instagram; Twitter). These comments perpetuated the notion that transgender women are power-hungry men in costume who want to invade in and prey on women’s spaces. An example was seen in the following statement:

So a guy with only average abilities among other men gets tired of being average and making no money in athletics and gets the bright idea of competing against women, as a woman. So he makes the great sacrifice, because he is not actually a woman in a male body, and transitions into a woman. (Washington Post)

The above comment alluded to some “great sacrifice,” presumably referring to giving up one’s manhood. There is implicit misogyny associated with the idea of “sacrificing” one’s identity as a man or male. This comment also suggested that a man with “average abilities” would be able to succeed in elite women’s sport: an argument that paints assigned-female-at-birth (AFAB) athletes as weak and inferior to assigned-male-at-birth (AMAB) athletes. As such, there was a perpetuation of the notion that transgender women are predatory men masquerading as women for the sake of a trophy.

The argument of “cheating” is directly misogynistic, suggesting that the athletic advantages of transgender women are higher than cisgender women, even after bouts of hormone therapy; an often adopted stance that to date lacks theoretical support in the scientific literature.

### Comparison Lacking Nuance

In a similar fashion to the cheating accusations, a high proportion of comments demonstrated a lack of understanding of the physiology of transgender bodies in relation to cisgender bodies. Many commenters suggested that transgender women retain all of their pre-medical transition strength and abilities. Examples included: “He broke no records at all because he can’t win on an even playing field,” and “Wow, a guy in a wig and obviously psychological problems is stronger than a woman” (YouTube). These comments discredited and diminished the hard work and dedication to the sport of powerlifting accomplished by Mary Gregory. While Mary Gregory invested countless hours into her training while undergoing hormone replacement therapy, these accomplishments were weaponized against her by spectators.

Many individuals also seemed to not understand transgender identities, framing it as a choice or factor of life that should not and cannot be changed. One commenter posed: “So if I identified as a kid can I compete in T-Ball? I would dominate those chumps!!!” (YouTube). This comment addressed the issues of choice, transition possibilities, and competitive intention. Perhaps this comment meant to cast transgender identities as outrageous, just as identifying as a different age would be. This touched on the idea of choice, where one’s transness is seen as a conscious decision to “be trans.” Reducing transgender identity down to a “choice” diminishes the struggles, hardships, and violence faced by this community on the basis of gender. Additionally, by framing one’s so-called “choice” to be transgender as a competitive edge or a means to athletic success, perpetuates the notion that transgender women are cheating.

The reduction of transgender identities to “choice” also alluded to the argument of nature, whereby sex and gender are seen as inherently linked phenomena. This biological deterministic view of human “nature” is upheld by a gendered *status quo* within the larger society (Keddie et al., 2008). This *status quo* maintains a binary of the interchangeable sex and gender labels, with an assignment of rigid roles and prescriptions of behavior. With sport’s binary sex categories, it is no surprise that such a *status quo* is not only enforced, but socially perpetuated through transphobic rhetoric and exclusion of transgender athletes.

## Discussion

The purpose of this research was to examine the virtual violence and online discourse faced by transgender powerlifter, Mary Gregory, following the nullification of her results by the Powerlifting Federation. Online comments were analyzed using reflexive thematic analysis and five themes were generated, including, the invalidation of gender identity, dehumanization, crude and derogatory language, accusations of cheating, and comparison lacking nuance.

In interpreting the collective themes generated, it is suggested that the virtual violence experienced by Mary Gregory within powerlifting cyberculture, on the basis of her transgender identify, is a form of gender-based violence, or violence directed at individuals directly linked to their gender identity, gender expression and/or perceived gender. This study supports the growing body of literature on gender-based violence in sport (Lang et al., 2021), and specifically gender-based violence in online sporting spaces (Kavanagh et al., 2019). This study adds to this previous research by highlighting the violence experienced by transgender powerlifter Mary Gregory on the basis of her transgender identity. More specifically, the themes generated in this study highlight experiences of virtual gender-based violence exclusive to transgender individuals in sport. To capture these experiences, and stemming from the study findings, we propose the use of the abbreviation GBV-T* to refer to gender-based violence again trans* individuals. The term trans*—asterisk included—represents individuals across a spectrum of identities whose gender is different than that assigned at birth. The word trans* is used to describe the broad spectrum of cisgender non-conforming individuals, including non-binary, transgender, and gender non-conforming identities (Killermann, 2019). While cisgender describes individuals who identify with their sex assigned at birth (Lucas-Carr and Krane, 2011; Killermann, 2019), trans* people have no obligation to medically transition as a requirement of their identity.

The concept of gender-based violence experienced specifically on the basis of trans* identity (GBV-T*) supports previous research beyond sport, reporting that the majority of trans* individuals face some form of physical, sexual, and/or verbal assault on the basis of gender identity, gender expression and/or perceived gender within their lifetimes (Stotzer, 2009). Transgender people are among one of the most stigmatized and discriminated social groups in society, and are vulnerable to systemic inequities, as well as violence on the basis of their gender identities and expression (Grossman and D’Augelli, 2006; Symons and Hemphill, 2006). Research provides evidence of the wide range of violence that trans people experience, including but not limited to sexual assault, systemic discrimination, and the targets of microaggressions, such as misgendering, deadnaming, and perpetuating fallacious transphobic rhetoric (Wirtz et al., 2020), all examples of gender-based violence against trans* people (GBV-T*) found in this research in the online comments directed at Mary Gregory.

Based on the findings of this study, it is suggested that the perpetuation of GBV-T* and transphobic belief within powerlifting cybercultures may be the product of three factors. First, shared beliefs surrounding trans* athletes between members of the cyberculture. Similar themes of GBV-T* were found across every social media platform analyzed. This suggests that there is no advancement of knowledge or clear voices of empathy toward trans* lifters within these spaces. Additionally, the personalization of social media apps may contribute to this “echo chamber” on the basis of shared belief. Social media apps tend to show content of related interest to its users, leading to the echo chamber phenomenon (Du and Gregory, 2017). Posts with transphobic rhetoric-heavy comment sections may be shared more between individuals with transphobic beliefs, who are then more likely to comment and repeat the cycle of virtual GBV-T*.

The presence of shared beliefs contributes to the second factor that perpetuates this echo chamber: the lack of education about the social issues of equity and diversity in the specific context of powerlifting. Many shared beliefs perpetuate false ideas about trans* identity and physiology. In some cases, the expression and perpetuation of false ideas may have been due to the threat of masculinity. Previous research has reported that masculinity threats among cisgender heterosexual (cishet) men were related to “traditional attitudes” toward gender identity and roles (Kosakowska-Berezecka et al., 2016). The presence of trans* women within traditionally cishet spaces, such as powerlifting, can be classified as a threat to masculinity due to her challenge of both hegemonic gender roles and identities (Türkoğlu and Sayılan, 2021). In other cases, this may not be the direct fault of the cyberculture’s members, but rather a representation of the larger society’s shortcomings in education and awareness. Previous studies have shown that a greater awareness of social issues, by means of social movements, can be a catalytic factor in progressive policy change—as was the case for women’s rights movements (Htun and Weldon, 2012). In those cases, policy change represented a shift in *status quo* and social attitudes as a result of a push in knowledge and awareness.

Finally, the lack of self-edification can be explained by the third factor: the lack of repercussions both from social media platforms and powerlifting federations. It is easy to uphold a *status quo*, even one that is founded on prejudice, when there are no punishments for those who maintain that system of oppression. The “gendered *status quo*” found in sport is the reproduction of that found in society at large (Keddie et al., 2008). As such, there are no reparations for individuals impacted and outcast by the hegemony of gender, but rather the opposite. It is those impacted individuals, such as trans* individuals and athletes, who face the consequences and trauma of existing outside of the *status quo*. It then becomes the responsibility of the maintainers of this hegemony to make equitable change. Such actors in the maintenance of this gendered *status quo* of transphobia in powerlifting cybercultures are both social media platforms as well as powerlifting federations themselves. If each were to have strict athlete and spectator guidelines for interpersonal conduct, perhaps the normalization of transphobia an GBV-* would be questioned.

The online commentary targeting Mary Gregory specifically, but more broadly pertaining to trans* athletes was often coupled with statements that attempted to present these interactions as jokes or attempted humor. Lockyer and Savigny (2020) suggest, humor is recognized as a tool that is adopted in order to normalize and/or trivialize gender-based discrimination, while Cole (2015) suggests that such actions are implemented to neutralize a sense of threat. The violence that targeted Mary Gregory online included comments that were subtle and hidden through humor, or overt but coupled with attempts at humor; such methods serve to reduce the likelihood of these interactions being recognized as violent. Violent interactions mixed with the inclusion of “jokes” online are recognized to act as a mask to the severity of the interaction (Kavanagh et al., 2019).

Interestingly, while the study used an inductive process when analyzing the data, generated themes resemble aspects of microaggressions faced by individuals specifically identifying as LGBT (Nadal et al., 2010). Nadal and colleagues (2010) developed a taxonomy describing the types of microaggressions specifically based on sexual orientation and transgender identity. Categories of microaggressions included: (1) heterosexist and transphobic language, (2) affirmation of heteronormativity and cisnormativity through language and behavior, (3) homogenizing the LGBT experience, (4) exotifying dehumanizing microaggressions made toward LGBT individuals, (5) discomfort and disapproval of LGBT rights, (6) denying transphobia and heterosexism, (7) assumptions that all LGBT individuals are hypersexual or sexually deviant, and (8) denying one’s own transphobic and heterosexist beliefs/behaviors/language (Nadal et al., 2010). Though this study focused specifically on the trans* athlete experience, there are striking similarities such as transphobic language used against trans* individuals, the affirmation of cisnormativity through language, homogenization of the LGBT experience, dehumanization of LGBT people, lack of support for LGBT people and their rights, and denying cissexism in both prevalence and effects (Nadal et al., 2010). While the remaining three categories within the taxonomy had been present in the assessed comment sections, they were not explicitly present within the selected comment analyzed in this research. Nevertheless, there are clear connections between the *status quo* of transphobia within society at large, as represented by the microaggression taxonomy, and that of powerlifting cybercultures.

The actions of powerlifting subculture members put powerlifting federations in the spotlight. The findings of this study depict the nature of GBV-T* faced by trans* powerlifters for their membership within respective subcultures of powerlifting. Federations act as the foundation of these communities, setting the tone for equitable narratives, or lack thereof. By banning trans* athletes, the transphobic *status quo* and system of oppression against trans* athletes are upheld. On the other hand, a federation that favors inclusivity and equity for *all* members would open conversations that include trans* athletes, rather than silence them. We propose it is the responsibility of powerlifting federations to invest in research and development into equitable solutions for all lifters facing axes of oppression. It is also their responsibility to invest in community outreach programs for those who have been systematically marginalized and discriminated against by their policies: past and present. Of these solutions may include diversity seminars and training as a mandatory module for membership within their federations. Such a solution would address the lack of education within the powerlifting community that has resulted in the perpetuation of harmful GBV-T* narratives surrounding trans* athletes.

## Conclusion

The present study exposed the experience of gender-based violence against trans* powerlifter Mary Gregory, as a first-look into the social climate of powerlifting cybercultures. The results are interpreted to suggest violence and harm discourses toward Mary Gregory on the basis of her identity as a trans* individual. The radicalization of Mary Gregory’s identity produced a number of examples of virtual gender-based violence recurrent throughout the data. It is suggested that while Mary Gregory’s actions in the pursuit of athletic excellence and participation would be considered a non-issue for cisgender athletes, her existence threatened the gender/sex binary on which federated sport is founded on. Such a binary has only worked to perpetuate transphobic discourses within virtual powerlifting spaces, as found through this netnography and the themes generated in this research.

A significant contribution of this research is the development of the term GBV-T* to represent violence directed at victims directly linked to their gender identity, gender expression and/or perceived gender, and experienced by individuals across a spectrum of identities whose gender is different than that assigned at birth. We refer to the perpetuation of such violence as GBV-T*.

There are multiple directions recommended for further research in the field of GBV-T* in powerlifting, and in sport generally. In order to address equity and inclusion for trans* athletes in sport, there must be greater attention to the experiences of trans* athlete both within physical and virtual sporting cultures. While cisgender athletes with histories of performance-enhancing drug use are able to compete in drug-free powerlifting competitions, there must be research comparing their advantages to those of trans* athletes, as well as cisgender athletes without performance-enhancing drug histories. There should also be research assessing the impact of different types of advantages including but not limited to: physiological, socioeconomic, and geographical. The expectation of these proposed research directions is to clarify whether the exclusion of trans* athletes is quantified through data, or rather an extension of society’s gendered *status quo*. At the same time, there should be endeavors into the development of sport policy that creates sporting spaces inclusive of *all* athletes. This will exist in relation to the development of trans* athlete care programs that allow success interpersonally, with coaches, and with federations.

Future research should specifically examine the prevalence of gendered-based violence against trans* athletes across a far wider variety of platforms and temporal frames of reference. This paper as a single case highlights the hostility toward trans* powerlifter Mary Gregory as an individual example. Future studies should consider questions and methodologies that enable exploration of interactions online that capture broader commentary surrounding trans* athlete inclusion in order to demonstrate a more comprehensive understanding of the complexity and depth of online experiences of harm. Giles (2016) refers to the use of conversational analysis (CA) to explore discursive practices. Adopting such methods may generate the structural characteristics of threads (or online social commentary). There is also room to learn more about the impact of this virtual narrative on the victims or targets of such violence.

Ultimately, in order to shift away from the current GBV-T* experiences and transphobic standards of belief, in addition to further research, endeavors toward equity for all athletes, trans* and cisgender, should be taken up by powerlifting federations to ensure safety and inclusion for all athletes.

## Data Availability Statement

The original contributions presented in the study are included in the article. Further inquiries can be directed to the corresponding author/s.

## Ethics Statement

As this study relies purely on public archival data, no formal research ethics board approval was required. Several steps were taken to ensure the ethical considerations of a netnography research design. As there was no direct interaction or communication between researchers and “participants,” there was no requirement of consent in the use of archival data.

## Author Contributions

RT-T and AM conceptualized and designed this study. RT-T collected and analyzed the data. All authors contributed to the interpretation of the data, writing, and reviewing the manuscript.

## Conflict of Interest

The authors declare that the research was conducted in the absence of any commercial or financial relationships that could be construed as a potential conflict of interest.

## Publisher’s Note

All claims expressed in this article are solely those of the authors and do not necessarily represent those of their affiliated organizations, or those of the publisher, the editors and the reviewers. Any product that may be evaluated in this article, or claim that may be made by its manufacturer, is not guaranteed or endorsed by the publisher.

## References

[B1] Braun V. Clarke V. (2019). Reflecting on reflexive thematic analysis. *Qual. Res. Sport Exerc. Health* 11 589–597. 10.1080/2159676X.2019.1628806

[B2] Cleland J. Dixon K. Kilvington D. (2020). *Online Research Methods in Sport Studies.* London: Routledge.

[B3] Cole K. K. (2015). “It’s like she’s eager to be verbally abused”: Twitter, trolls, and (en) gendering disciplinary rhetoric. *Fem. Media Stud.* 15 356–358. 10.1080/14680777.2015.1008750

[B4] Colliver B. (2021). “Not the right kind of woman”: transgender women’s experiences of transphobic hate crime and trans-misogyny,” in *Misogyny as Hate Crime* eds Zempi I. Smith J. (Abingdon: Routledge), 213–227. 10.4324/9781003023722-11

[B5] Du S. Gregory S. (2017). “The echo chamber effect in Twitter: does community polarization increase?,” in *Proceedings of the Studies in Computational Intelligence*, Vol. 693 Cham. 10.1007/978-3-319-50901-3_30

[B6] Frederick E. Sanderson J. Schlereth N. (2017). Kick these kids off the team and take away their scholarships: facebook and perceptions of athlete activism at the University of Missouri. *J. Issues in Intercoll. Athl.* 10 17–34.

[B7] Giles D. C. (2016). Observing real world groups in the virtual field: the analysis of online discussion. *Br. J. Soc. Psychol.* 55 484–498. 10.1111/bjso.12139 26916617

[B8] Grossman A. H. D’Augelli A. R. (2006). Transgender youth: invisible and vulnerable. *J. Homosex.* 51 111–128. 10.1300/J082v51n01_06 16893828

[B9] Htun M. Weldon S. L. (2012). The civic origins of progressive policy change: combating violence against women in global perspective, 1975–2005. *Am. Polit. Sci. Rev.* 106 548–569. 10.1017/s0003055412000226

[B10] Jones B. A. Arcelus J. Bouman W. P. Haycraft E. (2016). Sport and transgender people: a systematic review of the literature relating to sport participation and competitive sport policies. *Sports Med.* 50 1857–1859. 10.1007/s40279-016-0621-y 32710429

[B11] Jones B. A. Arcelus J. Bouman W. P. Haycraft E. (2017). Barriers and facilitators of physical activity and sport participation among young transgender adults who are medically transitioning. *Int. J. Transgend.* 18 227–238. 10.1080/15532739.2017.1293581

[B12] Kavanagh E. Jones I. Sheppard-Marks L. (2016). Towards typologies of virtual maltreatment: sport, digital cultures and dark leisure. *Leis. Stud.* 35 783–796. 10.1080/02614367.2016.1216581

[B13] Kavanagh E. Litchfield C. Osborne J. (2019). Sporting women and social media: sexualization, misogyny, and gender-based violence in online spaces. *Int. J. Sport Commun.* 12 552–572. 10.1123/ijsc.2019-0079

[B14] Kavanagh E. Litchfield C. Osborne J. (2021). “Virtual technologies as tools of maltreatment: Safeguarding in digital spaces,” in *Routledge Handbook of Athlete Welfare*, ed. Lang M. (Abingdon: Routledge), 221–230. 10.4324/9780429201745-25

[B15] Keddie A. Mills C. Mills M. (2008). Struggles to subvert the gendered field: issues in masculinity, rurality and class. *Pedagogy Cult. Soc.* 16 193–205. 10.1080/14681360802142161

[B16] Killermann S. (2019). Defining LGBTQ+: A Guide to Gender & Sexuality Terminology. The Safe Zone Project. Available online at: https://thesafezoneproject.com/defining-lgbtq-a-guide-to-gender-sexuality-terminology/

[B17] Kilpatrick D. G. (2004). What is violence against women? Defining and measuring the problem. *J. Interpers. Violence* 19 1209–1234. 10.1177/0886260504269679 15534326

[B18] Kosakowska-Berezecka N. Besta T. Adamska K. Jaśkiewicz M. Jurek P. Vandello J. A. (2016). If my masculinity is threatened I won’t support gender equality? The role of agentic self-stereotyping in restoration of manhood and perception of gender relations. *Psychol. Men Masc.* 17 274–284. 10.1037/men0000016

[B19] Kozinets R. (2010). *Netnography: Doing Ethnographic Research Online.* Thousand Oaks, CA: SAGE Publications.

[B20] Krane V. (2014). “Inclusion to exclusion: sport for LGBT athletes,” in *Routledge International Handbook of Sport Psychology*, eds Schinke R. McGannon K. R. Smith B. (London: Routledge), 238–247.

[B21] Lang M. Meraert L. Arnaut C. Vertommen T. (2021). Gender-based violence in sport: prevalence and problems. *Eur. J. Sport Soc.* 1–22. 10.1080/16138171.2021.2003057 [Epub ahead of print].

[B22] Litchfield C. Kavanagh E. Osborne J. Jones I. (2018). Social media and the politics of gender, race and identity: the case of Serena Williams. *Eur. J. Sport Soc.* 15 154–170. 10.1080/16138171.2018.1452870

[B23] Lockyer S. Savigny H. (2020). Rape jokes aren’t funny: the mainstreaming of rape jokes in contemporary newspaper discourse. *Fem. Media Stud.* 20 434–444.

[B24] Lucas-Carr C. Krane V. (2011). What is the T in LGBT? Supporting transgender athletes through sport psychology. *Sport Psychol.* 25 532–548. 10.1123/tsp.25.4.532

[B25] MacPherson E. Kerr G. (2021). Sport fans’ perspectives of public shaming of professional athletes on social media. *Qual. Res. Sport Exerc. Health* 13 146–165. 10.1080/2159676x.2020.1836505

[B26] Nadal K. Rivera D. Corpus M. (2010). “Sexual orientation and transgender microaggressions in everyday life: experiences of lesbians, gays, bisexuals, and transgender individuals,” in *Microaggressions and Marginality: Manifestations, Dynamics, and Impact*, ed. Sue D. W. (New York, NY: John Wiley & Sons), 217–240.

[B27] Ohlert J. Vertommen T. Rulofs B. Rau T. Allroggen M. (2021). Elite athletes’ experiences of interpersonal violence in organized sport in Germany, the Netherlands, and Belgium. *Eur. J. Sport Sci.* 21 604–613. 10.1080/17461391.2020.1781266 32524909

[B28] Parent S. Vaillancourt-Morel M. P. (2020). Magnitude and risk factors for interpersonal violence experienced by Canadian teenagers in the sport context. *J. Sport Soc. Issues.* 45 528–544. 10.1177/0193723520973571

[B29] Reid E. Duffy K. (2018). A netnographic sensibility: developing the netnographic boundaries/social listening boundaries. *J. Mark. Manag.* 34 263–286. 10.1080/0267257x.2018.1450282

[B30] Sailors P. R. (2020). Transgender and intersex athletes and the women’s category in sport. *Sport Ethics Philos.* 14 419–431. 10.1080/17511321.2020.1756904

[B31] Sanderson J. Frederick E. Stocz M. (2016). When athlete activism clashes with group values: social identity threat management via social media. *Mass Commun. Soc.* 19 301–322. 10.1080/15205436.2015.1128549

[B32] Stotzer R. L. (2009). Violence against transgender people: a review of United States data. *Aggress. Violent Behav.* 14 170–170. 10.1016/j.avb.2009.01.006

[B33] Symons C. Hemphill D. (2006). Transgendering sex and sport in the gay games. *Sport Sex. Queer Theory* 1 109–128.

[B34] Tamminen K. A. Poucher Z. A. (2020). “Research philosophies,” in *The Routledge International Encyclopedia of Sport and Exercise Psychology: Theoretical and Methodological Concepts*, Vol. vol.1 eds Hackfort D. Schinke R. (Abingdon: Routledge).

[B35] Tominey C. (2019). *Transgender Athletes in Women’s Sport are as Unfair as East German drug Cheats, says Sharron Davies.* Available online at: https://www.telegraph.co.uk/news/2019/07/11/transgender-athletes-womens-sport-unfair-east-german-drugs-cheats/ (accessed on July, 11 2019)

[B36] Türkoğlu B. Say?lan G. (2021). How is masculinity ideology related to transprejudice in Turkey: the mediatory effect of femmephobia. *Psychol. Sex.* 13, 86–100.

[B37] Vertommen T. Veldhoven N. Woulters K. Kampen J. Brackenridge C. Rhind D. (2016). Interpersonal violence against children in sport in the Netherlands and Belgium. *Child Abuse Negl.* 51 223–236. 10.1016/j.chiabu.2015.10.006 26516053

[B38] Villareal D. (2019). *Inside the Dispute Between USA Powerlifting and a Minnesota Trans Woman Athlete.* Out Sports. Available online at: https://www.outsports.com/2019/10/31/20939582/usa-powerlifting-transgender-ban-minnesota-jaycee-cooper-larry-maile-joanna-harper (accessed October 21, 2019).

[B39] Willson E. Kerr G. Stirling A. (2021). Prevalence of maltreatment amongst Canadian national team athletes. *J. Interpers. Violence.* 10.1177/08862605211045096 [Epub ahead of print]. 34549664 PMC9554369

[B40] Wirtz A. L. Poteat T. C. Malik M. Glass N. (2020). Gender-based violence against transgender people in the United States: a call for research and programming. *Trauma Violence Abuse* 21 227–241. 10.1177/1524838018757749 29439615

